# Do adolescents with long-term illnesses and disabilities have increased risks of sports related injuries?

**DOI:** 10.1186/s40621-017-0112-0

**Published:** 2017-05-01

**Authors:** Kwok W. Ng, Jorma Tynjälä, Pauli Rintala, Sami Kokko, Lasse Kannas

**Affiliations:** University of Jyvaskyla, PO Box 35 (L), 40014 Jyvaskyla, Finland

**Keywords:** Disability, Chronic disease, Safety promotion, Physical activity, Organised sports, Health behaviours

## Abstract

**Background:**

The aim of this study is to examine the rates of sports related injuries in adolescents based on the severity of their long-term illnesses or disabilities (LTID). Few injury prevention strategies in sports and health promotion have explored disaggregation by disability.

**Methods:**

Data obtained from the 2014 Finnish Health Behaviour in School-aged Children survey (*n* = 3716, mean age = 14.8, SD = 1.03) were grouped into adolescents with and without LTID. A further indicator or severity was determined when adolescents reported their LTID affected their participation (affected LTID). Odds ratio (95% CI) were used to determine the associations between sports related injuries and LTID, daily moderate to vigorous physical activities (MVPA), being a sports club member, physical competence, and family encouragement, after controlling for age, gender and family affluence.

**Results:**

One in four adolescents (25%) reported to have LTID and one in eight adolescents (12.5%) reported sports injuries. The odds for adolescents with chronic conditions, functional and learning difficulties was the highest (OR 3.55, CI = 2.3–5.4) for overall injuries, when compared with adolescents without LTID. Adolescents with affected LTID (OR = 2.08, CI = 1.5–2.9) were more likely to report medically attended injuries than adolescents without LTID. Sports-related injuries (OR = 0.33, CI = 0.1–0.8) were lower in adolescents with affected LTID than those without LTID after adjusting for personal and environmental factors.

**Conclusions:**

Taking part in sport clubs increases the risk of sports related injuries in adolescents with and without LTID, but not with affected LTID. Few adolescents with affected LTID participate in sports clubs and were less likely to report the most serious type of injury to be from sports. These results could be used for devising sports based injury prevention and health promotion strategies for children with LTID.

## Background

Taking part in physical activities (PA) during adolescents is essential in the fight against obesity, to gain physiological and mental health benefits, as well as opportunities for youth social engagement in organised sport settings (WHO, 2010). This is even more important in adolescents with long-term illnesses or disabilities (LTID), since regular participation in PA can reduce the onset of secondary conditions to existing LTID (Rimmer et al. 2012). However, a critical consideration that researchers, professionals, parents, and individuals interested in promoting PA for health is the heightened risk of injuries through sports (Mitchell et al. 2010).

Recent meta-analysis of 15 studies with a total sample size of 9,581,553, including 83,286 adolescents with LTID, reported more injuries than their counterparts without LTID and the difference increases as the children get older (Shi et al. 2015). In addition, adolescents who reported more LTID conditions were at even greater risks of medically attended injuries (Raman et al. 2007). Numerous studies have reported the higher threat of sports related injuries among adolescents with LTID than the general participation (Shi et al. 2015). However, it is not clear if this is the same for all adolescents with LTID or if it is more common in specific types of conditions. Studies have reported injuries in adolescents with autism and history of seizures are more common than other conditions (Ramirez et al. 2004; Ramirez et al. 2009). Yet, the fear of injuries are commonly reported among adolescents with LTID (Bloemen et al. 2015) thus acts as barriers for PA participation (Pittet et al. 2009). Other modifiable factors that may influence the risk of injuries include individual physical condition, proprioception as well as physical competence (Emery 2003). Injury prevention programs targeted towards improved conditioning and balance training have increased exponentially between the 1970s and 2009 (Klugl et al. 2010). As such, the perceptions of physical competence are associated with levels of PA (Ng et al. 2016a) and may confound its associations with injuries.

Another contextual factor for exposure to sports related injuries is the environment they are in. Since young adolescents are under the care of their parents, the known risks of injury through sport may induce protective behaviours by the parents. Parents may feel it necessary to prevent injury by excluding their children with LTID from PA participation. Another environment that modifies injury risk at international level is the country. Clinical data provided by the Finnish Institute of Health and welfare (THL) indicates that injuries from sports are the 2nd most common form of emergency department hospitalisation between 10 and 14 year olds. The rate of injuries also increases during these ages and reaches a peak at the age of 15 (Mattila et al. 2004). Through international comparative non-clinical data, the association between the inequality gap, as measured by family affluence, and prevalence of medically treated injuries was the highest in Finnish boys in the international 2010 (Currie et al. 2009) and 2014 reports (Inchley et al. 2013). Similarly, studies have also reported the link between high family affluence and greater amounts of physical activity (Borraccino et al. 2009). Therefore, it was not surprising that data collected from the cross-national Health Behaviours in School-aged Children (HBSC) study revealed that just over half of medically treated injuries were through sporting activities (Pickett et al. 2005). Since the bottom end of the equity gap for PA is the smallest in Finland (Chzhen et al. 2016), it is important to study the associations in sports and injuries in Finland as this could negate the influence of family affluence. Therefore the purpose of this study is to investigate sports related injuries in relation to the severity of LTID, PA levels, sports club membership, physical competence, and family encouragement, after controlling for age, gender and family affluence. To proceed with this purpose, there are two parts to this study. The first part was to categorise adolescent with LTID and define the association with medically attended injuries. Following this, contextual factors were examined in relation to sports related injuries.

## Methods

### Sampling and procedures

This study is part of the Finnish version of the Health Behaviours in School-aged Children (HBSC) study. Adolescents (*n* = 3716) from schools all around Finland were included to generate a national representative sample consisted of only 13 and 15 year olds, with a response rate of 84% of 13 year olds and 86% of 15 year olds. The procedures of data collection followed the international HBSC study protocols (Roberts et al. 2009). Selected schools were randomly selected through two levels, regions of Finland (Capital, South, Middle, North), and type of area (urban, sub-urban, rural) using proportion probably size. The primary sampling unit was the school. According to the international HBSC protocol, schools had to come from the Finnish general education school setting, therefore private schools and special educational needs schools were not included. School principals agreed to permit pupil participation through passive consent and the study had ethical approval by the National Board of Education in Finland at the beginning of the study. The survey was completed anonymously and voluntarily through pen and paper during the spring of 2014.

### Instruments

#### Long term illness or disabilities

Two items from the chronic conditions short questionnaire (CCSQ) (Dale & Marsh 1993) were used in this analysis to identify the health condition and impairment domains of the International Classification of Functioning, Disability and Health (ICF) (WHO 2001); “Do you have a long-time disability, illness or medical condition (such as cerebral palsy, diabetes, arthritis or an allergy) diagnosed by a doctor?” Responses were “Yes” and “No. The second item; “Does your long-term illness or disability affect your participation in school?” had response categories of “I do not have long-term illness or disabilities”, “yes”, and “no” was also included. Severity of disability was defined at three levels; (1) no disabilities (no LTID), (2) disabilities, but does not affect their participation in school (LTID), (3) disabilities with affecting participation in school (affected LTID). Both items have been developed from population surveys aimed at measuring disabilities (McDougall & Miller 2003).

#### Health conditions

Following from completion of CCSQ, three categories of health conditions were measured; chronic conditions, learning difficulties and functional difficulties (Table 1). Items were from two item sets. One item set was for functional and learning difficulties, “Tick only one box that best describes you, using the following descriptors; Do you have difficulty…. (a) in seeing, even with glasses, (b) in hearing, even with a hearing aid, (c) in speaking fluently, (d) in moving, (e) in handling objects, (f) in breathing, for example, shortness of breath, (g) in remembering things, (h) in concentrating, (i) in sitting still during lessons.” Response categories were used to measure severity included (1) No difficulties, (2) Difficulties do not affect functioning, (3) Difficulties affect functioning somewhat, (4) Difficulties affect functioning a lot, (5) Difficulties affect functioning very much. In line with the coding of the ICF (Selb et al. 2015), any items a-f with severities from 3 to 5 were grouped as functional difficulties. Any items g-i with severity from 3 to 5 were grouped as learning difficulties.

**Table 1:** The types of long-term illnesses or disabilities (LTID) and Chi-square test of independence in the proportion that LTID affects the individual’s participation

	LTID	Affected LTID	n^a^	*p*-value*
	(%)	(%)		
None	88.0	12.0	125	0.158
Chronic condition	81.7	18.3	546	0.052
Allergies	83.1	16.9	391	0.688
Asthma	75.9	24.1	187	0.002
Diabetes	75.0	25.0	64	0.051
Epilepsy	29.4	70.6	17	<.001
Joint pains	69.4	30.6	216	<.001
Learning difficulties	72.2	27.8	252	<.001
Remembering	69.8	30.2	149	<.001
Concentrating	70.0	30.0	180	<.001
Sitting still	69.0	31.0	113	<.001
Functional difficulties	67.9	32.1	218	<.001
Breathing	63.7	36.3	113	<.001
Handling objects	44.4	55.6	27	<.001
Hearing	52.9	47.1	17	0.001
Moving	47.6	52.4	63	<.001
Seeing	67.9	32.1	53	0.001
Speaking	66.2	33.8	65	<.001
Overall	83.7	16.3	942	

*LTID* Long term illnesses or disabilities

* Pearson’s Chi-square test of independence

^a^ Columns are dominator for %. Overall totals do not add up due to multiple conditions and difficulties per individual case

Health conditions are recognised as an independent domain in the ICF (WHO 2001). In the first item of the CCSQ, there were examples of LTIID “(such as … diabetes, arthritis or an allergy)”. These examples are not actual functional difficulties, yet seem to influence the way respondents self-identify themselves to have LTID. In previous rounds of HBSC, more than half of adolescents with LTID had conditions that were not functional (Ng et al. 2014). To measure severity of the condition in line with the examples in the CCSQ and the ICF qualifiers, the measurement of frequency of symptoms related to diabetes, arthritis, allergy and epilepsy were used. These items were used an extension the HBSC health complaints checklist (Haugland & Wold 2001), “How often have you had the following symptoms over the past 6 months? Tick one box for each symptom. (a) epileptic seizure symptoms, (b) allergy related symptoms, (c) diabetic symptoms caused by low or high blood sugar levels, (d) asthmatic symptoms such as wheezing, (e) severe joint pains (other than growing pains). Response categories included, (1) Almost daily, (2) More than once a week, (3) Approximately once a week, (4) Approximately once a month, (5) Less or never.” Symptoms frequency of at least once a week was grouped as ’Chronic Conditions’. Observations of each LTID groups were combined to indicate co-existence of chronic conditions, functional difficulties and learning difficulties.

#### Sports injuries

Measures of medically attended injuries were derived from the 1988 Child Health Supplement to the U.S. National Health Interview Survey (Scheidt et al. 1995) and have been well validated and reliability ascertained from earlier international studies (de Looze et al. 2012; Molcho et al. 2006). The item included; “How many times during the past 12 months have you been injured so that you have been treated by a doctor or a nurse?” Responses included (1) I have not been injured over the last 12 months, (2) Once, (3) Twice, (4) Three times, (5) Four or more times.

Location of injury item has been used in the HBSC study in 1997 (Pickett et al. 2005) with the question; “Where were you when the most serious injury happened? Please tick the most appropriate box.” Possible responses included, (1) I have not been injured in the past 12 months, (2) At home / in yard, (3) School during school hours, (4) School after school hours, (5) At a sports facility or field (not school), (6) In the street / road / car park, (7) Somewhere else.

Injury activity item, with a similar methodological history as the location of injury item (Pickett et al. 2005); “What were you doing when the most serious injury happened? Please tick the most appropriate box.” Responses options included, (1) I have not been injured in the past 12 months, (2) I was riding a bike, (3), I was playing or training for sports or a hobby, (4), I was walking/running (excluding training for team sports or competitive sport), (5), I was driving or riding a moped car or other motor vehicle, (6), I was fighting, (7) I was doing paid or unpaid work, (8), I did something else.

For the analyses in this study, several steps were taken to create a variable named, sports related injuries. First, pupils that indicated that they had at least one medically attended injury in the last 12 months were included in the sports related injury analysis. There were three groups of sports related injuries. Pupils that selected “at a sports facility or field (not school)” from the injury location item were used to identify serious injuries at a sports facility. Pupils that selected “I was playing or training for sports or a hobby” from the injury activities item were used to identify serious injuries while doing sports. Pupils that selected both “at a sports facility or field (not school)” and “I was playing or training for sports or a hobby” were used to identify serious injuries while doing sports at sports facility.

#### Physical activities

##### Moderate to vigorous physical activities

The self-reporting measure of at least 60 min of moderate to vigorous physical activity was used (Prochaska et al. 2001). It consists of a single item asking the pupils how many days (0–7) in the past week they were physically active for 60 min or more (cumulative). Two groups were created based on the moderate to vigorous physical activity (MVPA) recommendations for adolescents set at every day (daily) and less than 7 days (not daily) (WHO, 2010). The measure has moderate validity and good reliability in Finnish adolescents (Vuori et al. 2005).

##### Sports club

Pupils were asked whether they belong and practice in a sports club or not. The response options included “not a member”, “a member but not active”, “a member and active”, grouped as non-member, inactive, and active members, respectively. The nature of the analysis was to examine active members in an environment related to sports injuries, therefore the inactive members were grouped together with not members. The item has been used in national monitoring surveys for many years (Vuori et al. 2004).

##### Physical competence

A single item that measured perceived physical competence was worded in the following way, “how good are you at sports compared to others the same age as yourself?” Response categories included “among the best”, “Good”, “Average”, “Below average”. Responses with average and below average were grouped into “low competency” group and good and among the best into the high competency group. The reliability of the item is sufficient in Finnish adolescents (Lintunen et al. 1995).

##### Family support

During a typical week: how often your parents (or guardians)… “encourage you take part in physical activities or sports”. Response categories were, “never”, “rarely”, “sometimes”, “often”, “very often”. In the analysis, frequent family support was considered when responses were “often” and “very often”, and the infrequent support included “never”, “seldom”, and “sometimes”. Reliability and validity studies are lacking on this particular question, although almost half of the adolescents (48.0%) responded frequent family support.

#### Family affluence scale

The Family Affluence Scale III (FAS III) is a composite indicator of self-reported socio-economic status that includes six items of material wealth (number of holidays taken during the last year, dishwasher in the house, family car ownership, having one’s own bedroom, number of bathrooms in the house, computer ownership). A collective sum score was created to then divide into three equal groups of low, medium and high family affluence, as replicated in an international validation study of the FASIII (Torsheim et al. 2015).

### Analysis

The data file was cleaned for missing data. T-tests of injury frequency and MVPA were not statistically significant from completed data. Imputation of missing data was not necessary and the cases were removed. Descriptive statistics were used to report the prevalence of LTID, LTID classification, and severity of LTID by gender. Frequencies and cross-tabulations were used to compare the prevalence of the types of conditions between the groups of adolescents without, with and with affected LTID. Reported injuries in the last year were also compared through Chi-square test of independence statistics. Statistical significance was set at p < 0.05 with IBM SPSS 20.0.

Associations between LTID severity and injury types were examined using multiple logistic regression to estimate adjusted odds ratio (OR) and its 95% confidence intervals (CI). Separate models were conducted for three types of injuries, (i) serious injuries at a sports facility, (ii) serious injuries while doing sports, iii) serious injuries while doing sports at sports facility. Included into these models were MVPA, sports club membership, physical competence, family encouragement, FAS, gender and age.

## Results

A total of 3665 students (mean age = 14.8 years, SD = 1.03) were included in the 2014 Finnish HBSC study. There were slightly more girls (51%) than boys (49%) in the sample. One in four (*n* = 942/3665) reported to have a LTID, of which 16% (*n* = 154/942) or 4% (*n* = 154/3665) of the total reported to have LTID that affects their daily participation. This latter group is referred to as the group with affected LTID. Table 1 contains information on the measures used in this study based on whether or not the individual reported a medically attended injury or not. The denominator for the percentages was the sample size for each value of interest.

In spite of the fact the adolescent reported to have LTID, information from the scales used was not able to identify the condition in 13% of the sample. Half of the adolescents with LTID (*n* = 465/942) reported to have only chronic conditions, while a third (*n* = 306/942) reported coexistence of chronic conditions with functional difficulties, chronic conditions with learning difficulties, or chronic conditions with functional difficulties and with learning difficulties. Less than a one in seven adolescents with LTID (*n* = 125/942) reported no functional difficulties, chronic conditions or learning difficulties listed in the survey questionnaire (Table 2). These proportions did not vary by age group, gender or family affluence (FAS III). There were no differences in the number of sports club members between adolescents with (45.5%) and without LTID (41.7%), yet there were significantly (p < .001) less members that had affected LTID (27.6%).

**Table 2:** Descriptive statistics of no injuries and at least one injury in last 12 months

	No injuries	At least one injury	n	*p*-value*
	(%)	(%)		
LTID				<.001
No LTID	64.0	36.0	2723	
LTID	53.3	46.7	788	
Affected LTID	46.1	53.9	154	
Gender				<.001
Boy	57.8	42.2	1772	
Girl	64.1	35.9	1893	
Age category				0.654
13	60.7	39.3	1759	
15	61.4	38.6	1906	
Family Affluence Scale III				<.001
Low	67.4	32.6	482	
Medium	63.4	36.6	2104	
High	54.0	46.0	1016	
Sports Club Member				<.001
Not Member	69.6	30.4	2048	
Member	50.4	49.6	1477	
Daily MVPA				<.001
Not Daily	64.4	35.6	2879	
Daily	49.1	50.9	786	
Physical Competence				<.001
Low	68.2	31.8	1568	
High	55.8	44.2	2097	
Family Encouragement				<.001
Infrequent	64.7	35.3	1909	
Frequently	57.1	42.9	1756	

*LTID* Long term illnesses or disabilities, *MVPA* Moderate to vigorous physical activity

* Pearson’s Chi-square test of independence

### Rates of medically attended injury

A total of 1448 students (39%) reported one or more medically attended injuries during the 12 months prior to completing the survey (Table 3). These injuries included sports related injuries as well as other types. Over half of sports club members (54%) reported medically attended injuries. Adolescents with affected LTID reported significantly more medically attended injuries (M = 2.22, CI:1.99–2.45) than their peers with LTID (M = 1.82, CI:1.74–1.90) in the last 12 months. Adolescents without LTID reported significantly less number of medically attended injuries (M = 1.58, CI:1.55–1.74) in the last 12 months.

**Table 3:** Distribution of the groups of long-term illnesses or disabilities (LTID) and LTID status (%)

	n	LTID	Affected LTID^a^	*p*-value*
None	125	14.0	9.7	0.158
Functional	7	0.8	0.6	0.882
Learning	22	2.3	2.6	0.814
Learning and Functional	17	1.3	4.5	0.005
Chronic	465	53.4	28.6	<.001
Chronic and Functional	93	8.8	15.6	0.009
Chronic and Learning	112	11.5	13.6	0.464
Chronic, Learning, and Functional	101	8.0	24.7	<.001
Total (n)	942	788	154	

n (sample size) - denominator, *LTID* Long-term illnesses or disabilities

* Chi-square test of independence of LTID types and status

^a^ sum equals 99% since Affected LTID Column is rounded to 1 decimal point

### Sports related injury activity and location

Over half of the adolescents that reported medically attended injuries stated that their most serious injury happened while playing or training (52.9%). However, significantly (p < .001) fewer adolescents with affected LTID (29.0%) stated playing or training in sports as the activity of the most serious injury. In addition, serious injuries from walking or running were generally low (3.8%), and there were no statistical differences between LTID groups.

A third of adolescents that reported the most serious injury took place at the sports facility at the time of injury (34.7%). Nevertheless, there were significantly (*p* = .018) less adolescents with affected LTID (19.7%) that were injured at a sports facility. Other common places for injuries for adolescents with affected LTID included at home (23%) and at school during school hours (21%) (Table 4).

**Table 4:** Injury characteristics (%) for each long-term illnesses or disabilities status

	No LTID^a^	LTID^a^	Affected LTID^a^	*p*-value*
Place when injury happened				<0.001
No Injury	60.2	49.1	44.2	
At home/yard	7.2	11.0	13.0	
School during school hours	5.6	5.7	11.7	
School after school hours	1.1	2.3	2.6	
Sports facility	13.5	18.4	11.0	
Street	5.3	4.4	7.8	
Other	7.0	9.0	9.7	
Missing cases	0.0	0.0	0.0	
Activity during injury				<0.001
No Injury	60.7	49.0	42.2	
Biking	3.1	2.5	3.9	
Playing or training	19.4	24.4	13.6	
Walking/ running	1.2	2.4	3.9	
Riding/ driving in a car	2.2	2.9	6.5	
Fighting	0.9	0.5	4.5	
Work	0.4	0.5	1.3	
Other activity	11.6	17.6	23.4	
Missing cases	0.4	0.1	0.6	
Injured and sports club membership				<0.001
Not member	56.3	51.9	68.2	
Member	40.2	43.3	26.0	
Missing cases	3.4	4.8	5.8	

LTID (Long-term illnesses or disabilities)

No LTID (no reported LTID), LTID (yes, LITD), affected LTID (LTID and affects participation)

*Pearson’s Chi-square test of independence

^a^ 2723 adolescents without LTID, 788 adolescents with LTID, and 154 adolescents with affected LTID as the denominators

### Relationships between disability status and injury

The odds for reporting a medically attended injury is double for adolescents with affected LTID, and one and a half times for adolescents with LTID than their peers without LTID (Table 5). Furthermore, adolescents with symptoms from chronic conditions were between 1.6 and 3.6 times more likely to report medically attended injuries. Adolescents with chronic conditions, learning difficulties and functioning difficulties had the highest odds of almost four times when compared to adolescents without LTID.

**Table 5:** Adjusted odds ratio (OR) and 95% confidence intervals from binary logistic regressions for the association between medically attended injuries in the past 12 months and LTID status and groups

	n	OR	Lower CI	Upper CI
LTID Status				
No LTID	2723	1.000		
LTID	788	**1.559**	**1.328**	**1.830**
Affected LTID	154	**2.082**	**1.502**	**2.885**
Groups of LTID				
None	2848	1.000		
Functional	7	1.352	0.302	6.054
Learning	22	1.503	0.647	3.490
Learning & Functional	17	0.984	0.363	2.667
Chronic	465	**1.605**	**1.317**	**1.956**
Chronic & Functional	93	**2.190**	**1.445**	**3.318**
Chronic & Learning	112	**1.679**	**1.150**	**2.451**
Chronic, Learning & Functional	101	**3.553**	**2.335**	**5.407**

Bold text showing statistically significant results

*LTID* Long-term illnesses or disabilities

n (Sample size), OR (Adjusted Odds Ratio), Lower CI (Lower 95% Confidence Interval), Upper CI (Upper 95% Confidence Interval)

Adolescents without LTID were three times more likely to report injuries at a sports facility during practice or training for sports than adolescents with affected LTID (Table 6). The associations between adolescents with LTID and injuries while training for sports and injuries at a sports facility were significantly greater than adolescents without LTID. In addition, the association between injuries at a sport facility were six times higher for sports club members than non-members. Adolescents with high perceived physical competence and frequent encouragement by parents reported greater risks in sports related injuries.

**Table 6:** Adjusted odds ratio (OR) and 95% confidence intervals from binary logistic regressions for sports related injuries

	Injury from training for sports (*n* = 3513)				Injury at sports facility (*n* = 3525)				Injury from training for sports at facility (*n* = 1507)			
	n	OR	Lower CI	Upper CI	n	OR	Lower CI	Upper CI	n	OR	Lower CI	Upper CI
LTID Status												
No LTID	2620	1.000			2630	1.000			1044	1.000		
LTID	749	**1.273**	**1.032**	**1.571**	750	**1.394**	**1.098**	**1.771**	378	1.076	0.805	1.438
Affected LTID	144	0.864	0.507	1.472	145	1.032	0.551	1.932	85	**0.333**	**0.142**	**0.777**
Sports Club Member												
Not Member	2041	1.000			2048	1.000			718	1.000		
Member	1472	**4.112**	**3.363**	**5.029**	1477	**6.267**	**4.848**	**8.102**	789	**6.553**	**4.808**	**8.932**
MVPA												
Not Daily	2760	1.000			2768	1.000			1096	1.000		
Daily	753	**1.335**	**1.089**	**1.636**	757	**1.369**	**1.092**	**1.717**	411	1.139	0.863	1.504
Physical Competence												
Low	1516	1.000			1520	1.000			547	1.000		
High	1997	**1.769**	**1.432**	**2.186**	2005	**2.167**	**1.663**	**2.822**	960	**2.243**	**1.633**	**3.081**
Family Support												
Infrequent	1832	1.000			1836	1.000			719	1.000		
Frequent	1682	**1.317**	**1.095**	**1.585**	1689	**1.440**	**1.160**	**1.787**	788	**1.482**	**1.137**	**1.930**

Data from only those that reported most serious injuries after controlling for Age, Gender and Family Affluence

Bold text showing statistically significant results

*LTID* Long-term illnesses or disabilities, *MVPA* Moderate to vigorous physical activities

n (Sample size), OR (Adjusted Odds Ratio), Lower CI (Lower 95% Confidence Interval), Upper CI (Upper 95% Confidence Interval)

## Discussion

### More conditions and severity, and more likelihood of injuries

The first part of this study examined medically attended injuries among adolescents with and without LTID from a national representative sample in Finland. Adolescents with LTID are more likely to report medically attended injuries and the odds increases to just over two times if the LTID affects their daily participation. In addition, of the three LTID classifications, adolescents with chronic conditions had significantly increased odds of reporting medically attended injuries. These findings concur with existing literature as rates of injuries in adolescents with specific conditions more common than other conditions (Ramirez et al. 2004; Ramirez et al. 2009).

The findings from this study continue to provide evidence that adolescents with LTID are at greater risk of injuries than adolescents without LTID (Shi et al. 2015; Mattila et al. 2004). The design of the study is original as it used data collected from general school systems to make comparisons between LTID groups possible. In many cases, pupils who attend these schools have lower severity of disabilities than those that would require special educational support in special schools. Despite these population differences, the adolescents in this study still had greater odds of injuries over their peers without LTID.

In all cases of the various LTID classifications, there were greater odds of reporting injuries. Other studies on injuries of children with chronic conditions, functional disabilities and intellectual disabilities have reported similar results (Shi et al. 2015; Ramirez et al. 2004). Although caution when interpreting the results is needed, as often there is a lack of information regarding the types of LTID. In an attempt to overcome these limitations, items related to health conditions and functional difficulties were incorporated into the 2014 Finnish data collection round. Functional impairments may put the adolescent at risk for injuries in general, such as knocking into things and falling down with visual impairments, unable to hear warning sounds for adolescents with hearing difficulties, as well as delayed movement to a safe place when there is a danger. Comorbidity can increase the odds up to three fold when adolescents reported to have chronic conditions, learning and functional difficulties. This may be related to the way the adolescents’ process information about their environment and avoid vulnerable situations.

### Contextual factors associated with sports related injuries

The second part of the study was to investigate sports related injuries in relation to degrees of LTID, sports club membership, physical competence, and family encouragement, after controlling for, gender, age and family affluence. Other studies have claimed that athletes with LTID that take part in organised sports have lower exposure to injury (Ramirez et al. 2009). Through the performed analyses, we complemented these earlier findings. Adding to the literature, in Ramirez’s study (Ramirez et al. 2009), the researchers followed special education interscholastic league for the course of a season. In recent years, there has been an increase in overall PA in adolescents with LTID (Ng et al. 2016b) and the amount of activities targeted for children that attend special education in Finland (Mononen et al. 2014). However, studies like these often fail to report whether children that attend these activities are part of the mainstream school system, hence providing addition information to this phenomena.

While there was weak evidence to suggest that there are differences in the amounts of physical activity, sports club membership and physical competence between adolescents with and without LTID, there seem to be a difference if the adolescent has affected LTID. Previous research suggests that a low exposure to physical activities shows low levels of sports related injuries (Raman et al. 2007), and in our study, approximately half of adolescents with affected LTID were likely to report their main injury to have taken place during sports. Adolescents with affected LTID may have functional difficulties, learning difficulties or chronic conditions that affect their participation and these elevated risks of injuries may be from serious non-sports related events.

In the opposite direction of the low exposure and low risk association, sports club members were between four and seven times more likely to report sports related injuries. This was expected as the exposure to activities is greater than non-members. Furthermore, adolescents with high physical competence are twice likely to experience sports related injuries. High physical competence, is not an indicator of actual performance, but linked to high confidence to perform (Kipp & Weiss 2013). Athletes may then be at a greater risk of sport injuries.

After adjusting for sports club membership, and daily physical activities, adolescents with affected LTID were less likely to report sports related injuries than adolescents without LTID. In other research, non-organised sports produces nearly twice as many injuries as in the organised sports setting (Zaricznyj et al. 1980), and this may be an incentive for people at risk of injuries to attend sports clubs rather than to pursue their own activities as recent trends have shown (Ng et al. 2016b). Sport clubs have a responsibility to adhere to safety promotion guidelines. More research on the effectiveness of such guidelines after taking into considering the inclusion of disabilities in sports club is needed. Creating an inclusive environment for athletes to support each other regardless of disabilities may encourage more membership among affected LTID adolescents. Yet, the steps for clubs to do this can take many years to develop as the many club settings are run by volunteers (Geidne et al. 2012; Kokko 2014).

### Strengths and limitations

This study has a number of strengths and limitations that the reader may like to consider when interpreting the results of the study. The HBSC survey has collected national representative data since 1983. No statistical weights were applied since, no formal register of disability statistics in schools is currently available. The sampling procedures do not include special schools, where children with special educational support needs often attend. It is not clear if the classes chosen to complete the questionnaire are representative of the school for the prevalence of LTID. Data collection was anonymous, reducing response bias, but sacrifices class level statistical models. Construct validity of some items remain unknown, for example, the wording of the family encouragement item was originally in Finnish and subsequently translated into English. Contextual meaning may be different from the literal meaning in this item. In addition, the data does not allow for interpretation of the actual meaning of ‘serious injury’ as perceived among the respondents. There were no items about milder injuries, other locations for sports related injuries, or details of the types of injuries that took place in the sports facilities. In addition, although respondents indicated the place and activity of their most serious injury, there is a lack of clarity in whether the injuries only during sports club activities or other times, such as recreation or in school. Also, lack of specificity on physical activities do not allow differentiation between activities attended during physical therapy, physical education or sport club activities. As such, it could limit the precision of safety promotion messages. Adolescents self-reported their difficulties, and this perception can be hard to quantify when measured against medical standards. Another explanation for the relatively high proportion of adolescents who reported LTID (25%) was the omission of the words, “please do not include learning disabilities” to be in line with other national surveys such as England, France, Ireland and Portugal (Sentenac et al. 2013). Therefore, it was important to utilise a functional, learning, and symptom based measures to correspond to the ontology of the biopsychosocial model of disability, especially in the associations to injuries. A dedicated survey for disabilities would have been useful to know more about the difficulties, although space, anonymity, and other logistics prevented this.

## Conclusions

Taking part in regular physical activities can improve overall health, but it comes at a risk for injuries in adolescents. It is even more important for adolescents with long-term illnesses or disabilities to consider the risks with the advantages, since inactivity in these children can lead to more health complications. Personal and environmental factors may influence adolescents’ reporting of sports related injuries. More strategies are needed to reduce injury rates from sport settings while increasing rates of PA in adolescents with LTID.

## Abbreviations

CCSQ: Chronic conditions short questionnaire
FAS: Family affluence scale
HBSC: Health Behaviour in School-aged Children study
ICF: International Classification of Functioning, Disability and Health
LTID: Long-term illnesses or disabilities
MVPA: Moderate to vigorous intensity physical activity
PA: Physical activities
